# GeCoNet-Tool: a software package for gene co-expression network construction and analysis

**DOI:** 10.1186/s12859-023-05382-1

**Published:** 2023-07-11

**Authors:** Junyao Kuang, Kristin Michel, Caterina Scoglio

**Affiliations:** 1 Department of Electrical and Computer Engineering, Kansas State University, Manhattan, KS 66506 USA; 2 Division of Biology, Kansas State University, Manhattan, KS 66506 USA

**Keywords:** co-expression network, Missing value, Pearson correlation

## Abstract

**Background:**

Network analysis is a powerful tool for studying gene regulation and identifying biological processes associated with gene function. However, constructing gene co-expression networks can be a challenging task, particularly when dealing with a large number of missing values.

**Results:**

We introduce GeCoNet-Tool, an integrated gene co-expression network construction and analysis tool. The tool comprises two main parts: network construction and network analysis. In the network construction part, GeCoNet-Tool offers users various options for processing gene co-expression data derived from diverse technologies. The output of the tool is an edge list with the option of weights associated with each link. In network analysis part, the user can produce a table that includes several network properties such as communities, cores, and centrality measures. With GeCoNet-Tool, users can explore and gain insights into the complex interactions between genes.

## Background

A gene co-expression network is helpful for analyzing and predicting gene functions and regulations [1, 2]. A gene co-expression network is composed of nodes and edges, in which nodes represent genes and edges represent co-expressed gene pairs [3, 4]. In the past decade, high throughput technologies (such as single-cell RNA sequencing) have enabled biologists to measure gene expression levels under various conditions [5, 6]. To study the relationships between genes, some researchers employ dimension-reduction algorithms such as PCA, UMAP, and t-SNE [7–10] to visualize genes in 2D or 3D space. However, analyzing co-expression data that encompasses diverse conditions can be challenging, particularly when missing values are present [6, 11–40].

In our previous work [1], we developed a data processing scheme to construct a gene co-expression network for *Anopheles gambiae*. The experimental results demonstrated that the proposed approach is effective in studying gene functions and patterns, even when dealing with different experimental technologies and many missing values. Building upon this prior work, we developed an integrated tool–GeCoNet-Tool for gene co-expression network construction and analysis.

## Implementation

**Fig. 1:**
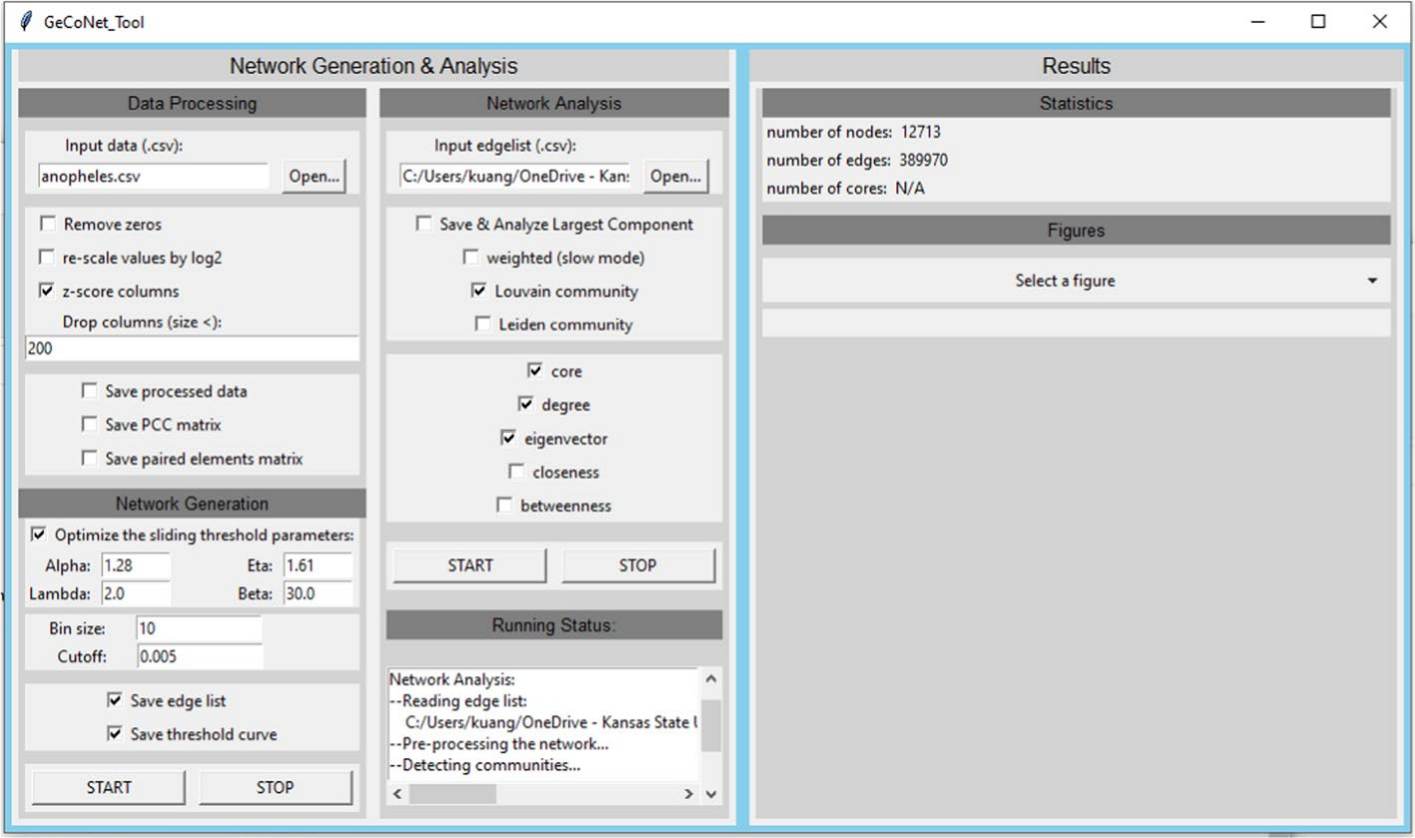
The GeCoNet_Tool user interface

GeCoNet-Tool is an open-source package that combines gene co-expression network construction and network analysis. This tool is an implementation and improvement of our previous work [1], which presented a scheme for studying gene expression data across a large number of conditions. GeCoNet-Tool is an executable file written in Python with a user-friendly graphical interface that facilitates configuration for different data types (as shown in Fig. 1). The process of using GeCoNet-Tool can be split into two independent parts: network construction and network analysis.

### Data processing and network generation

To construct a gene co-expression network, the user needs to input a gene co-expression matrix (.csv format), in which rows represent *N* genes and columns represent *M* experimental conditions. GeCoNet-Tool allows users to process the input data with different options, depending on the data type. For example, users can choose to remove zeros, re-scale expression values by log2, or normalize columns by z-score if the input data are obtained through RNA-seq [41]. Additionally, users can choose to save the processed data in table format.

GeCoNet-Tool calculates the Pearson Correlation Coefficient (PCC) between each pair of genes based on the processed data. The PCC matrix is saved as an upper triangular matrix if the user chooses to save the PCC matrix [42, 43]. In our previous work [1], we observed that the number of experimental conditions could significantly affect the PCC between two genes. Therefore, GeCoNet-Tool determines the number of paired elements between every pair of genes, and the user can save this data as an additional upper triangular matrix.

GeCoNet-Tool classifies the PCCs into different intervals based on the number of paired conditions of gene pairs. The user can specify the size of intervals (*Bin size*) in the GeCoNet-Tool interface. To select edges based on PCC value, the user also needs to input the cutoff value expressed as the chosen top percentage of all PCCs in a given interval (e.g., 0.005, 0.01, or 0.02), which is used to determine the sliding threshold by fitting the following curve:

1 $$\begin{aligned} f^{thres}(x)=\alpha -\frac{1}{\eta +\lambda e^{-\frac{x}{\beta }}}, \end{aligned}$$

where $\alpha$, $\eta$, $\lambda$, and $\beta$ are the four parameters that were fitted, and *x* is the number of paired elements. This equation provided a good trade-off between the accuracy of the fitting and the number of parameters to estimate [1]. Once the curve is fitted, the optimal parameters will be updated to the input boxes $\alpha$, $\eta$, $\lambda$, and $\beta$.

In order to obtain optimized parameters, GeCoNet-Tool automates the optimization of the four parameters instead of manual optimization as in our previous work [1]. The coefficient of determination (R-squared) of the fitted curve is shown in the *Running Status* box. The edges of the co-expression network are selected through the fitted curve based on the number of paired elements [1]. The user can construct networks with different edge densities by tuning the cutoff value.

We recommend using a cutoff value that can maintain the majority of the nodes connected while minimizing the number of edges. In general, increasing the cutoff value, decreases the edge density, which can impact the connectivity of nodes in a network. It is therefore advisable to choose a cutoff value that maintains the majority of connected nodes. To achieve this, the package should be executed multiple times using different cutoff values. This allows the user to observe the number of nodes and edges in each resulting network and select the cut-off value that contains the majority of nodes, while minimizing the number of edges. This approach ensures that the network retains its integrity while avoiding an excessive number of edges, which can impact downstream analyses.

Finally, users can save the list of edges in the co-expression network, along with the fitted threshold curve, for further analysis. The edge list and threshold curve are saved in the same folder as the input data.

### Network analysis

Once a network is constructed, various properties of the network can be analyzed through the second part of GeCoNet-Tool. GeCoNet-Tool allows users to produce the following network properties [44]:

- community: The community is defined as a subgraph that is highly connected internally and loosely connected to other subgraphs. GeCoNet-Tool allows users to detect communities through the Louvain and Leiden algorithms [45–47]. Users can customize the settings for community analysis by editing the original code (network_analysis.py), while the tool provides default settings for users who prefer to use them.
- core: The core of the network is obtained by repeatedly removing nodes with a degree less than k by starting with k = 1 and increasing k until no nodes are left in the network. The core genes are those with a degree = k (i.e., those removed during the last iteration) [48].
- degree: GeCoNet-Tool calculates node degree if the network is unweighted and calculates node strength if the network is weighted [44].
- eigenvector: GeCoNet-Tool calculates the eigenvector centrality, which is determined by the entry of the eigenvector corresponding to the largest eigenvalue of the adjacency matrix of the network [49].
- betweenness: GeCoNet-Tool calculates the betweenness centrality, determined by the number of shortest paths that pass through the node itself [50].
- closeness: GeCoNet-Tool calculates the betweenness centrality, which is based on the distances between nodes. Closeness centrality is the sum of the shortest path distance reciprocals of a node to all other nodes [51].

In the package, users can choose to analyze either the entire network or only the largest connected component and use either unweighted or weighted edges. GeCoNet-Tool generates a table in the output that contains all the selected properties. In addition, the package creates figures of the node degree distribution, community distribution, and core distribution.

## Results

The user can observe the running status of the GeCoNet-Tool through the *Running Status* window. At the same time, network statistics (such as the number of nodes, edges, and core nodes will be shown in the *Results* window as soon as the network is generated or analyzed.

**Fig. 2:**
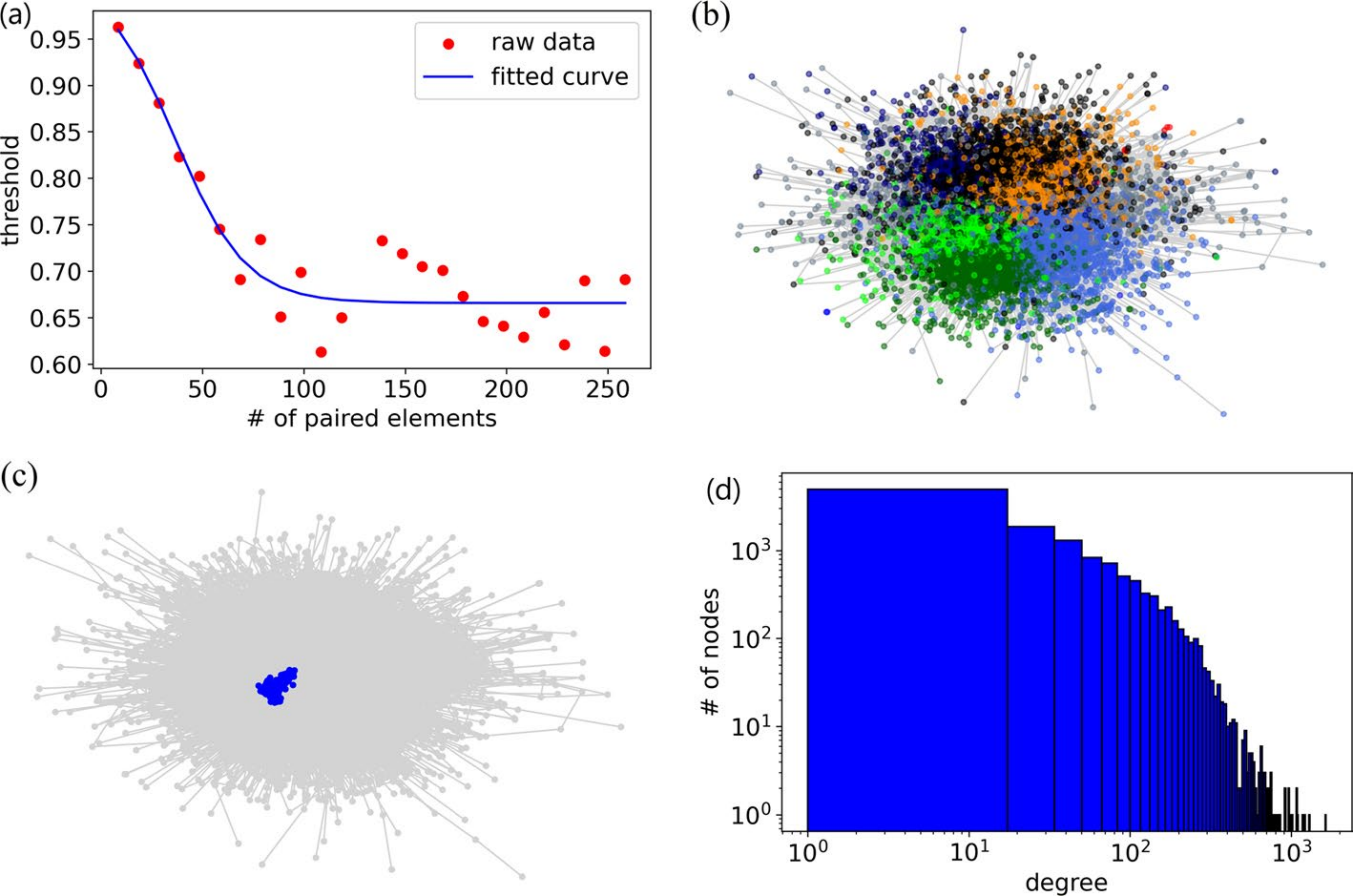
Examples of figure outputs from GeCoNet-Tool using *Anopheles gambiae* expression data.** a** The fitted threshold curve,** b** communities,** c** core nodes, and** d** degree distribution of the *An. gambiae* gene co-expression network

In the experiment, we provide the *Anopheles gambiae* gene expression data and generate a network with default settings (the data is publicly available through VectorBase (www.vectorbase.org) at the following URL: https://tinyurl.com/mr38a7hj). Figure 2a shows the sliding threshold with a cutoff value of 0.005, and the R-squared is 0.908, which suggests that the curve fits the raw data well. In practical applications, we suggest using smaller bin sizes and testing various cutoff values to generate a network that connects the majority of nodes while minimizing the number of edges.

In the second part, users can analyze the generated network and produce a table to store the properties of the network. In the *Anopheles gambiae* gene co-expression network, there are 12660 nodes, 389991 edges, and 164 core nodes. GeCoNet-Tool employs the force-directed algorithm Fruchterman-Reingold layout to visualize the community distribution (Fig. 2b) and core nodes (Fig. 2c). However, the layout shown in the results window is deterministic. Interested users are recommended to use interactive network visualization algorithms in Gephi [53] to show the generated network and properties. GeCoNet-Tool also generates node degree distribution as shown in Fig. 2d.

## Conclusion and future works

GeCoNet-Tool is a free and user-friendly research tool that offers a straightforward approach to network construction and analysis, without the need for coding expertise. The package is composed of two parts: (1) network construction and (2) network analysis. In the first part, pairwise relationships between nodes are evaluated using the PCC and the number of paired conditions. Users can choose from various expression data types and data processing options, such as removing zeros and log2-rescaling. In the second part, GeCoNet-Tool provides multiple tools for network analysis. For example, the community analysis will classify nodes into different communities to identify genes with similar biological functions.

In the *GeCoNet-Tool*, networks are currently constructed with the Pearson correlation coefficient. However, future updates may be expanded to other methods to assess gene co-expression, e.g., signed distance correlation, Spearman correlation, and mutual information, as suggested by recent studies [54]. This approach would offer users more flexibility in constructing co-expression networks that are tailored to their specific research requirements.

## Data Availability

Project name: GeCoNet-Tool. Project home page:https://github.com/KSUNetSE/GeCoNet-Tool. Operating system(s): Windows 10. Programming language: Python. License: Redistribution and use in source and binary forms, with or without modification, are permitted. The resources and data used during the current study are available in the GitHub repository, https://github.com/KSUNetSE/GeCoNet-Tool
